# The Function of the Hypothalamic–Pituitary–Adrenal Axis During Experimental Autoimmune Encephalomyelitis: Involvement of Oxidative Stress Mediators

**DOI:** 10.3389/fnins.2021.649485

**Published:** 2021-06-17

**Authors:** Svetlana Trifunovic, Ivana Stevanovic, Ana Milosevic, Natasa Ristic, Marija Janjic, Ivana Bjelobaba, Danijela Savic, Iva Bozic, Marija Jakovljevic, Katarina Tesovic, Danijela Laketa, Irena Lavrnja

**Affiliations:** ^1^Institute for Biological Research “Siniša Stanković”-National Institute of Republic of Serbia, University of Belgrade, Belgrade, Serbia; ^2^Medical Faculty of Military Medical Academy, Institute of Medical Research Belgrade, University of Defense, Belgrade, Serbia; ^3^Department for General Physiology and Biophysics, Faculty of Biology, University of Belgrade, Belgrade, Serbia

**Keywords:** multiple sclerosis, experimental autoimmune encephalomyelitis, hypothalamic–pituitary–adrenal axis, oxidative stress, cytokines

## Abstract

Multiple sclerosis (MS) is an inflammatory, demyelinating disease with an unknown origin. Previous studies showed the involvement of the hypothalamic–pituitary–adrenal (HPA) axis to susceptibility to autoimmune diseases, including MS, and its best-characterized animal model, experimental autoimmune encephalomyelitis (EAE). During MS/EAE, innate immune cells are activated and release cytokines and other inflammatory mediators, leading to a vicious cycle of inflammation. In response to inflammation, the activated HPA axis modulates immune responses *via* glucocorticoid activity. Because the mechanisms involving oxidative stress to the HPA axis are relatively unrevealed, in this study, we investigate the inflammatory and oxidative stress status of HPA axis during EAE. Our results reveal an upregulation of *Pomc* gene expression, followed by POMC and ACTH protein increase at the peak of the EAE in the pituitary. Also, prostaglandins are well-known contributors of HPA axis activation, which increases during EAE at the periphery. The upregulated Tnf expression in the pituitary during the peak of EAE occurred. This leads to the activation of oxidative pathways, followed by upregulation of inducible NO synthase expression. The reactive oxidant/nitrosative species (ROS/RNS), such as superoxide anion and NO, increase their levels at the onset and peak of the disease in the pituitary and adrenal glands, returning to control levels at the end of EAE. The corticotrophs in the pituitary increased in number and volume at the peak of EAE that coincides with high lipid peroxidation levels. The expression of MC2R in the adrenal glands increases at the peak of EAE, where strong induction of superoxide anion and malondialdehyde (MDA), reduced total glutathione (GSH) content, and catalase activity occurred at the peak and end of EAE compared with controls. The results obtained from this study may help in understanding the mechanisms and possible pharmacological modulation in MS and demonstrate an effect of oxidative stress exposure in the HPA activation during the course of EAE.

## Introduction

Multiple sclerosis (MS) is an idiopathic disease, with an origin that remains elusive. The causes that contribute to MS involve immune, genetic/epigenetic, and environmental factors. It is proposed that the autoimmune component underlies the onset of MS, with neuroinflammatory and neurodegenerative outcomes. Although it does not replicate every sequence of MS, experimental autoimmune encephalomyelitis (EAE) is the best-described animal model of MS and shares some features with the disease, mainly at histopathological and immunological levels. The initial period of EAE is characterized by the breakdown of the blood–brain barrier, followed by inflammatory cell infiltration in the white matter of the CNS. At the later stages, inflammation is accompanied by microglia and astrocyte activation, with ensuing demyelination and axonal loss (Bjelobaba et al., 2017, 2018).

Besides well-known immune-mediated demyelinating effects during MS, several studies pointed to and linked the endocrine system to the predisposition and severity of the disease (Huitinga et al., 2003; Gold et al., 2005; Melief et al., 2013). The endocrine system interacts with the nervous and immune system and elicits a response to homeostasis disturbance provoked by inflammation, stress, and/or infection (Smith and Vale, 2006; Procaccini et al., 2014). In this context, the critical player in maintaining physiological homeostasis under these circumstances is the hypothalamic–pituitary–adrenal (HPA) axis. Basically, parvocellular neurons in the paraventricular nucleus of the hypothalamus secrete corticotropin-releasing hormone (CRH) at the median eminence. CRH is transported through the hypothalamic–hypophysial portal blood vessels to the anterior pituitary, where it stimulates the release of adrenocorticotropic hormone (ACTH) from corticotrophs (Dallman et al., 1987) to the systemic circulation (Smith and Vale, 2006; Herman and Tasker, 2016). At the level of adrenal glands, ACTH acts *via* melanocortin 2 receptor (MC2R) causing synthesis and secretion of the glucocorticoids (GCs) (Beuschlein et al., 2001) cortisol (dominant GC in humans) and corticosterone (dominant GC in rodents) into the bloodstream (Smith and Vale, 2006). GCs’ action in the target tissues depends on the expression of glucocorticoid and mineralocorticoid receptors and on the cellular metabolism of GCs mediated by 11β-hydroxysteroid dehydrogenase isozymes (Chapman et al., 2013). In the tissues, especially in the placenta or kidney, the biologically active forms of glucocorticoids—cortisol and corticosterone—are converted to the inactive forms, cortisone and 11-dehydrocorticosterone, respectively, by the action of 11β-hydroxysteroid dehydrogenase 2. On the other hand, another isozyme, 11β-hydroxysteroid dehydrogenase 1, expressed especially in the liver, brain, inflammatory cells, adipose tissue, and gonads, regenerates active forms of GCs (Chapman et al., 2013; Prevatto et al., 2017). The release of GCs from the adrenal glands in return suppresses the release of ACTH levels in the anterior pituitary and CRH levels in the hypothalamus, representing a typical homeostatic negative feedback control mechanism (George et al., 2006).

Previous studies linked the dysregulation of the HPA axis and susceptibility to autoimmune diseases. Specifically, it was proposed that modulation of HPA axis activity is associated with the predisposition, severity, and heterogeneity of MS (Grasser et al., 1996; Gold et al., 2005; Melief et al., 2013). During MS/EAE, innate immune cells are activated and cytokines and other inflammatory mediators, including prostaglandins that are well-known contributors of HPA activation, are released (Silverman et al., 2005; Bellavance and Rivest, 2014). In response to inflammation, the activated HPA axis modulates immune responses *via* the activity of GCs. Pertinent to this, during inflammatory processes, HPA activation represents an important protective mechanism, where GCs, the end products of the HPA axis, exhibit immunosuppressive effects on the inflammatory setup in MS/EAE. Therefore, cortisol is used as first-line therapy in treating MS relapses (Bjelobaba et al., 2017). However, excessive HPA stimulation and long-term therapy increase susceptibility to infection and have well-known effects on neuroinflammation and cognition, thus limiting the therapeutic use of GCs in MS (Goodin, 2014; dos Santos et al., 2019). Subsequent to increased inflammation in MS, excessive generation of reactive oxygen species (ROS) (Berg et al., 2004) due to deregulation of mitochondrial electron-transport chain activity, induced by inflammatory mediators, may provoke oxidative damage to a variety of cells (Miljković and Spasojević, 2013). Besides the increased generation of ROS, depletion of antioxidants additionally contributes to redox imbalance, leading to oxidative stress (Birben et al., 2012). While physiological levels of ROS have a fundamental role in maintaining the homeostasis of the HPA axis (Spiers et al., 2015; Prevatto et al., 2017), increased generation of ROS, in an inflammatory setup, induces hyperactivation in the HPA axis. In addition, ROS modulates the redox regulation of various intracellular signaling systems and enhances the susceptibility of various tissues to injury (Mittal et al., 2014; Brown and Griendling, 2015). The exact mechanisms linking oxidative stress to the HPA axis are relatively unidentified (Schiavone et al., 2013).

Therefore, the aim of this study was to investigate the inflammatory and redox status of the HPA axis during EAE, because a better understanding of the relations between the neuroendocrine and the immune system may yield potent paradigms for the treatment of autoimmune diseases, including MS.

## Materials and Methods

### Experimental Animals

In this study, we used 2-month-old female rats of Dark Agouti (DA) (RRID:RGD_21409748) strain from the local colony. Experimental procedures and animal care were performed in accordance with European Ethical Normative (Directive 2010/63/EU) on the protection of animals used for experimental and other scientific purposes and in accordance with national regulative given by the Animal Welfare Law of the Republic of Serbia (“Official Gazette of Republic of Serbia” No. 41/2009). The experimental protocols were approved by The Ministry of Agriculture, Forestry and Water Economy of the Republic of Serbia (permit no. 323-07-05970/2020-05). The reported results comply with the ARRIVE guidelines. The animals were kept under standard laboratory conditions, with 12 h dark/light cycle, at constant temperature (23 ± 2°C) and humidity (50–60%), where they had free access to laboratory chow and water *ad libitum*. In total, 72 animals were used for the experiments. The number of animals for EAE experiments was calculated using power calculations (G^∗^Power Freeware, Version 3.1.9.7). Based on an alpha error probability of < 0.05, a beta of < 0.95, and standard deviations, the minimal number of animals needed to analyze and detect difference is *n* = 6 per group resulting in an actual power of 0.955.

### Assessment of EAE and Tissue Sample Collection

**FIGURE 1:**
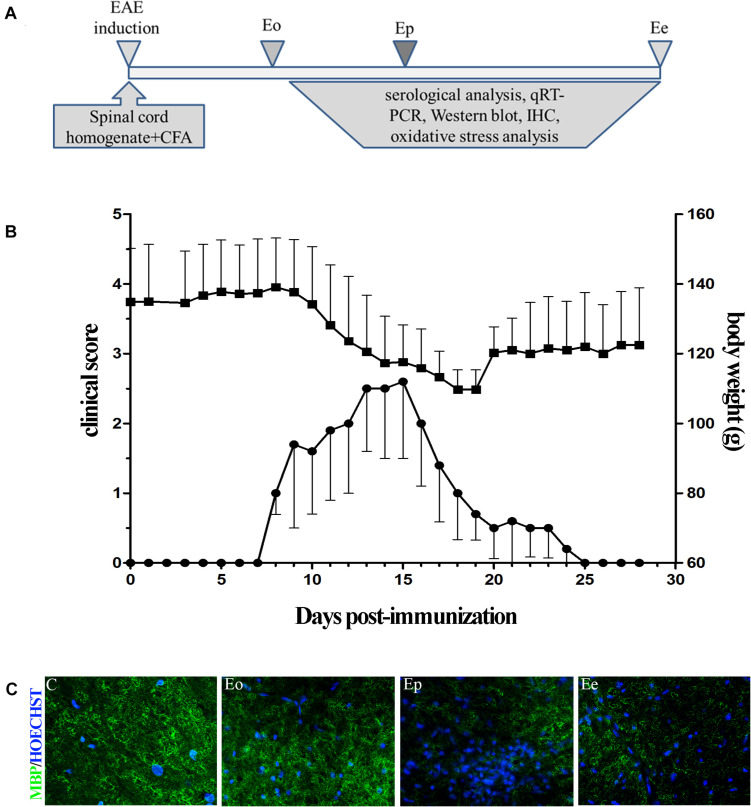
**(A)** Timeline of the experimental design. Rats were immunized with the homogenate of the spinal cord and CFA. The rats were sacrificed at the onset of the first symptom (Eo), peak of disease (Ep), and end of symptoms (Ee). **(B)** Time course of experimental autoimmune encephalomyelitis (EAE) symptoms and variation in the weight of the animals. Data have been expressed as mean ± SEM of daily measurements of each animal. **(C)** Inflammation was observed using Hoechst stain (blue), while demyelination was detected using the MBP antibody (green fluorescence).

The animals were immunized under carbon dioxide (CO_2_) anesthesia with a subcutaneous injection of encephalitogenic emulsion in the right hind hock. The encephalitogenic emulsion contained equal volumes of rat spinal cord homogenate (50% w/v in phosphate-buffered saline, PBS) and complete Freund’s adjuvant (CFA; Sigma, St. Louis, MO, United States), containing 1 mg/ml of *Mycobacterium tuberculosis*. Age-matched animals were anesthetized under the same conditions but were not immunized, and they were used as a control group. Animals from both experimental groups were monitored and weighed daily for 28 days after immunization. All animal experiments started at 10:00 a.m. every day. Disease severity was assessed by checking the neurological signs of EAE, following the subsequent scale: 0–unaffected; 0.5–reduced tail tone; 1–tail atony; 1.5–moderately clumsy gait, impaired righting ability, or combination; 2–hind limb paresis; 2.5–partial hind limb paralysis; 3–complete hind limb paralysis; 3.5–complete hind limb paralysis with forelimb weakness; 4–moribund state; and 5–death. For each time point, average disease score and weight were calculated. When animals had a disease score of 2.5 or higher, they were fed and given water by hand. The animals were euthanized by gradual asphyxia in a CO_2_ chamber at three time points representing three phases of the disease course: onset (Eo), peak (Ep), and end (Ee) of EAE. Afterward, blood was collected by cardiac puncture and the animals were perfused with 0.9% saline. The hypothalamic, spinal cord, anterior pituitary, and adrenal gland tissues were quickly dissected on ice. The adrenal gland capsule was removed and the left adrenal was processed for real-time PCR, while the right adrenal was used for oxidative stress analysis. The isolated tissues were further used for RNA isolation and quantitative real-time PCR, Western blot, immunohistochemistry/immunofluorescence, and oxidative stress evaluation. The investigators who performed the gene, protein, and biochemical studies were blinded to the experimental conditions. The timeline of the experiments is presented in Figure 1.

### Serological Analysis

#### Corticosterone Measurement

Corticosterone was measured in the control (*n* = 6) and EAE animals (*n* = 6/group) in serum acquired from blood samples obtained by cardiac puncture of at least six animals per group. Blood was centrifuged at 3,000 × *g* for 15 min, and all samples were stored at −80°C before determining corticosterone concentration using the commercially available Corticosterone Parameter Assay Kit (R&D Systems Inc., Minneapolis, MN, United States). The sensitivity of this kit is 0.047 ng/ml. The plate was read at 450 nm and wavelength correction was set to 570 nm. To determine the corticosterone level, calculation was performed using a four-parameter logistic curve fitting program.

#### Prostaglandin Levels

Serum PGE2 level was determined in the control (*n* = 6) and EAE animals (*n* = 6/group) from blood samples gathered by cardiac puncture. Blood was centrifuged at 3,000 × *g* for 15 min, and all samples were stored at −80°C before ELISA. The test was completed using a commercial PGE2 ELISA Kit (Invitrogen, Carlsbad, CA, United States) according to the manufacturer’s specifications. The absorbance was read at 405 nm and corrected at 570 nm. The concentrations of PGE2 obtained from the assay were determined by calculation using a four-parameter logistic curve fitting program.

### Quantitative Real-Time PCR Analysis

After isolation, hypothalamic, anterior pituitary, and adrenal gland tissues (*n* = 6/group) were stored at −80°C in RNAlater^®^ RNA Stabilization Solution (Ambion^TM^, Applied Biosystems by Thermo Fisher Scientific, Waltham, MA, United States) until all groups were collected. RNA was isolated using RNeasy Mini Kit (QIAGEN, Hilden, Germany) or RNAqueous^®^ Kit (Ambion^TM^, Applied Biosystems by Thermo Fisher Scientific), according to the manufacturer’s instructions. The concentration of RNA was assessed by measuring absorbance on Nanophotometer^®^ N60 (Implen, Munich, Germany) at 260 nm, while the purity of the samples was determined by *A*_260_/*A*_280_ and *A*_260_/*A*_230_ ratios. Furthermore, 1 μg of RNA was used for reverse transcription, done with High Capacity cDNA Reverse Transcription Kit (Applied Biosystems by Thermo Fisher Scientific). Quantitative real-time PCR analysis (qRT-PCR) was performed using 10 times diluted cDNA samples. QuantStudio^TM^ 3 Real-Time PCR System (Applied Biosystems by Thermo Fisher Scientific) was used for qRT-PCR with Power SYBR^TM^ Green PCR Master Mix (Applied Biosystems by Thermo Fisher Scientific) and primers were designed on specific targets. The primer sequences were as follows: *Pomc*, forward: AGAACGCCATCATCAAGAACG, reverse: AGGTCAGGTGCTCTCGCC; *Il1*β, forward: AAACAGCAATGGTCGGGACA, reverse: GTCCGGGAAG GCTGATTAGG; *Tnf*, forward: CCCCCATTACTCTGACCCCT, reverse: CCCAGAGCCACAATTCCCTT; *Nos2*, forward: ACACAGTGTCGCTGGTTTGA, reverse: AACTCTGCTGTT CTCCGTGG; *Nfe2l2*, forward: GACTTGGAATTGCCACCGC, reverse: CCTGTTCCTTCTGGAGTTGCT; *M2cr*, forward: TCCCACCATATCCCCACAGT, reverse: GGAGCGGGCAA GTAAGAACA; and *Gapdh*, forward: CAACTCCCTCAA GATTGTCAGCAA, reverse: GGCATGGACTGTGGTCATGA (all from Invitrogen, Carlsbad, CA, United States). The expression levels of the target genes were evaluated by comparing their *Ct*-values to *Ct*-values of *Gapdh* from the same sample (2^–Δ*Ct*^ method).

### Western Blot

Isolated anterior pituitary tissue (*n* = 6/group) was homogenized in RIPA buffer (50 mM Tris pH 7.5, 150 mM NaCl, 1% NP-40, 0.1% SDS, 10 mM EDTA, 10 mM EGTA, 0.5% Triton X-100) with protease inhibitors (Roche, Penzberg, Germany). Subsequently, the samples were sonicated two times for 5 s while kept on ice and centrifuged at 14,000 × *g* for 30 min at 4°C. The resulting supernatants’ protein concentration was assessed using the Micro BCA Protein Assay Kit, following the manufacturer’s instructions (Thermo Fisher Scientific, Rockford, IL, United States).

The samples were diluted in 4 × Laemmli sample buffer, incubated for 5 min at 95°C, and loaded onto 10% polyacrylamide gel. After electrophoresis, the resolved proteins were transferred to PVDF membranes (Immobilon-P transfer membrane, Millipore, Darmstadt, Germany) for 1 h at 100 V. The membranes were incubated in 5% bovine serum albumin (Sigma-Aldrich, Munich, Germany) in Tris-buffered saline (20 mM Tris, pH 7.4, 136 mM NaCl) with 0.05% Tween 20 (TBST), for 1 h at room temperature, to block unspecific binding. Afterward, the membranes were incubated with primary antibody (rabbit polyclonal antibody) directed against ACTH (obtained from Dr. A. Parlow, National Institute of Diabetes and Digestive and Kidney Diseases, National Hormone and Peptide Program, Torrance, CA, United States) and mouse monoclonal antibody against β-actin (Sigma-Aldrich, St. Louis, MO, United States) at 4°C overnight. On the next day, the membranes were washed three times with TBST and incubated with appropriate secondary antibodies conjugated with horseradish peroxidase (HRP) (purchased from Santa Cruz, CA, United States), for 2 h at room temperature. After washing three times in TBST, the membranes were incubated with SuperSignal^TM^ West Femto Maximum Sensitivity Substrate (Thermo Fisher Scientific, Waltham, MA, United States), and the bands were visualized on a FluorChem E Digital Imaging System (ProteinSimple, San Jose, CA, United States). ImageJ software package (RRID:SCR_003070) was used for the quantification of the signal and densitometric analysis. The optical density of the target protein was normalized to the optical density of β-actin of the same lane, and the results are expressed relative to the control group.

### Immunohistochemistry

Pituitary tissues (*n* = 6/group) were isolated and fixed in Bouin’s solution for 48 h, then dehydrated in a series of alcohol dilutions, immersed in xylene, and embedded in paraffin. The tissues were cut into 3-μm-thick paraffin sections, and labeling for ACTH was performed. The sections were quickly rinsed in PBS, and then unspecific binding was blocked by incubation of the tissues in 5% normal donkey serum (Sigma-Aldrich, Munich, Germany). The sections were incubated with rabbit polyclonal antibody directed against ACTH (purchased from Dr. A. F. Parlow, National Hormone and Peptide Program, CA, United States), overnight at 4°C. After washing in PBS, the sections were incubated with donkey anti-rabbit secondary HRP-conjugated antibody (1:250, Santa Cruz, Santa Cruz Biotechnology, CA, United States) for 2 h at room temperature. Immunoreactivity was visualized with 3,3′-S-di-amino-benzidine-tetrahydrochloride (DAB, Dako, Glostrup, Denmark) as substrate. The sections incubated without primary antibodies resulted in the absence of any specific reaction. After dehydration and clearing, the tissue was mounted using DPX Mounting medium (Fluka, Buchs, Switzerland). The slides were examined under a light microscope (Olympus, BX-51, Olympus, Japan) equipped with a microcator (Heidenhain MT1201, Heidenhain, Schaumburg, IL, United States). The stereological analyses were carried out using the new CAST (Computer Aided Stereology Tool) stereological software package (CAST software, Visiopharm, ver. 2.12.1.0, Hørsholm, Denmark), as previously described in detail (Trifunović et al., 2014). Briefly, using unbiased stereological analysis, the anterior pituitary volume and the volume density of ACTH cells were estimated using Cavalieri’s principle (Gundersen and Jensen, 1987). Every 20th section was analyzed to determine the stereological parameters of ACTH cells, i.e., about 24 sections for each gland. A fractionator/physical dissector design was used to estimate the number of ACTH cells, while the cell volume was calculated as equivalent to the total volume occupied by ACTH cells divided by their number. The micrographs are captured using a Zeiss Axiovert microscope (Carl Zeiss GmbH, Vienna, Austria).

#### Immunofluorescence

The spinal cord tissues were collected in order to perform immunofluorescence analyses. Spinal cords were fixed in 4% paraformaldehyde in 0.1 M PBS, pH 7.4 for 12 h at 4°C. Cryoprotection of the spinal cord was achieved by tissue immersion into the graded sucrose solutions (10–30% in 0.1 M PBS, pH 7.4). The lumbar parts were embedded in OCT (Sakura, Japan) in a cryomold. Frozen blocks were further stored at −80°C until sectioning. Coronal sections (20 μm thick) of the lumbar spinal cord were cut and placed on a SuperFrost glass slide. The sections were allowed to dry for 2 h at room temperature (RT) and stored at −20°C until staining. Sections were first rinsed in PBS and then incubated in normal donkey serum (10% solution in PBS; Santa Cruz Biotechnology, Santa Cruz, CA, United States) for 1 h at RT to block unspecific labeling. Incubation with primary antibody for myelin basic protein (MBP 1:100) (BioLegend 801703, RRID:AB_510039) was done overnight at 4°C. After washing in PBS, the sections were incubated with Alexa Fluor 488 fluorescent antibody. Nuclei were visualized with Hoechst nuclear staining. Sections incubated with appropriate secondary antibodies without the primary antibody were used as negative control. The sections were mounted in Mowiol (Calbiochem, Millipore, Darmstadt, Germany) and captured on Zeiss Axiovert fluorescent microscope (Zeiss, Graz, Austria).

### Parameters of Oxidative Stress

Pituitary and adrenal gland tissues were isolated and homogenized in RIPA buffer with protease inhibitors (Roche, Penzberg, Germany). Then, the samples were sonicated three times for 5 s and kept on ice. After centrifugation at 14,000 × *g* for 30 min at 4°C, the supernatants were collected and used for the determination of oxidative stress parameters: production of NO, O_2_^–^, malondialdehyde (MDA), and total glutathione content (GSH) and activity of antioxidative enzymes catalase, mitochondrial superoxide dismutase (SOD2), glutathione peroxidase (GPx), and reductase (GSR) as described previously (Bozic et al., 2015).

#### Determination of NO Production

NO production was determined using the Griess assay. Since NO is a volatile molecule, we measured the concentrations of its products, nitrites, and nitrates. Nitrates were first reduced into nitrites by metallic cadmium (Navarro-Gonzálvez et al., 1998), and then total nitrites were quantified spectrophotometrically by the Griess reaction. The Griess reagent was made of 1.5% sulfanilamide (Sigma-Aldrich, Munich, Germany) in 1 M HCl and 0.15% N-(1-naphthyl) ethylendiamine dihydrochloride (Fluka, Buchs, Switzerland) in distilled water. Known sodium nitrite concentrations were used to generate a standard curve by which the nitrite concentration in the samples was calculated. The results are expressed as mean nitrite concentration (μM/mg protein) ± SEM.

#### Superoxide Anion Radical

The method based on the reduction of nitroblue tetrazolium (NBT, Sigma-Aldrich, Munich, Germany) to monoformazan by O_2_^–^ was used for evaluating the concentration of O_2_^–^ in the samples. The product of this reaction was measured spectrophotometrically at 550 nm (Auclair and Voisin, 1985). The results are expressed as mean reduced NBT (μM/mg protein) ± SEM.

#### Malondialdehyde

The MDA concentration was evaluated using a spectrophotometric method (Villacara et al., 1989). The samples were incubated with thiobarbituric acid (TBA) reagent (15% trichloroacetic acid and 0.375% TBA, water solution, Merck, Darmstadt, Germany) at 95°C and pH 3.5. MDA formed a red product with the TBA reagent, and the absorbance was measured at 532 nm. The results are expressed as the mean concentration of MDA (nmol/mg protein) ± SEM.

#### Total Glutathione Content

Total glutathione content was determined using the DTNB-GSSG reductase recycling assay. The formation of 5-thio-2-nitrobenzoic acid (TNB), corresponding to total glutathione content in the sample, was measured spectrophotometrically at 412 nm (Anderson, 1985). Glutathione concentration in the samples was estimated from a standard curve created using known GSSG concentrations. The results are expressed as the mean concentration of glutathione (nmol/mg protein) ± SEM.

#### Glutathione Peroxidase Activity

GPx activity was indirectly measured using spectrophotometric determination of NADPH consumption mediated by GPx, as previously described (Bozic et al., 2015). The results are expressed as the amount of reduced NADPH per mg of total protein in the sample (mol NADPH/mg protein) ± SEM.

#### Glutathione Reductase Activity

GSR catalyzes the reduction of GSSG to GSH by the oxidation of the coenzyme NADPH to NADP^+^. A decrease in NADPH fluorescence was determined with excitation/emission of 360/460 nm. As a standard, we used 100 mM NAD^+^. The results are expressed as the extent of reduced NADPH (μmol NADPH/mg protein) ± SEM.

#### Mitochondrial Superoxide Dismutase Activity

The activity of SOD2 was measured spectrophotometrically by determining a decrease in the rate of the spontaneous epinephrine autoxidation at 480 nm. The kinetics of enzyme activity was measured in 50 mM carbonate buffer (pH 10.2, containing 0.1 mM EDTA, Serva, Feinbiochemica, Heidelberg, Germany), after the addition of 10 mM epinephrine and 5 mM KCN. The results are expressed as units of enzyme activity per mg of total protein (U/mg), where one unit represents the amount of enzyme required for 50% inhibition of autoxidation of epinephrine.

#### Catalase Activity

Catalase (CAT) activity was determined spectrophotometrically by measuring the absorbance of the colored complex formed between ammonium molybdate and H_2_O_2_ at 405 nm. The results are expressed as units of CAT activity per mg of total protein (U/mg), where one unit is the amount of H_2_O_2_ reduced per min (μM H_2_O_2_/min).

### Statistical Analysis

Statistical analysis was performed in GraphPad Prism 5 software (RRID:SCR_002798, GraphPad Software, La Jolla, CA, United States). No test for outliers was performed. All statistical analyses were performed by researchers who were unaware of the animals’ group. The results were tested for Gaussian distribution with the Kolmogorov–Smirnov normality test. If the groups had passed the normality test, the significance of the difference in the means between groups was calculated by one-way ANOVA (followed by Dunnett’s *post hoc* test). Otherwise, the Kruskal–Wallis test followed by Dunnett’s *post hoc* test was performed. Results are expressed as mean values ± SEM and values of *p* < 0.05 were considered to be statistically significant.

## Results

### Disability Score Coincides With Weight Loss in the Course of EAE

Female DA rats generated acute, monophasic disease after priming for EAE using homologous spinal cord tissue homogenate combined with CFA (Figure 1A). The first clinical deficit associated with EAE (flaccid tail) appeared between 8 and 10 dpi (Eo) and reached the peak of the symptoms (Ep) around 15 dpi. The development of neurological deficit was accompanied by a rapid and substantial (∼20%) weight loss. At 20 dpi, rats started to recover and gain weight. The absence of symptoms was evident at 25 dpi (Ee). All immunized rats developed a clinical score above 1, with a maximal severity score around 3 (Figure 1B). The clinical symptoms are followed by histopathological changes, inflammation, and demyelination. The most pronounced inflammation, judged by Hoechst staining, was observed at the peak of EAE and the observed inflammation-induced demyelination was seen by MBP staining (Figure 1C). Little inflammation/demyelination was observed at the onset and end of EAE.

### POMC and ACTH Showed Different Expression Patterns During EAE

**FIGURE 2:**
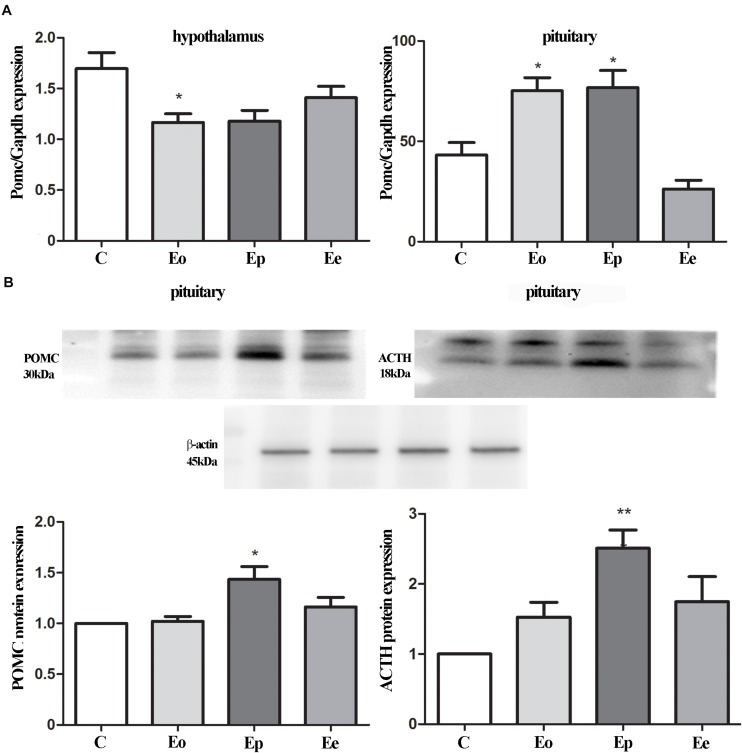
**(A)** POMC gene expressions were assessed by qRT-PCR in immunized (Eo, Ep, and Ee) and control animals (C–control), with Gapdh as an internal reference standard in the hypothalamus and pituitary. **(B)** Protein levels of POMC and adrenocorticotropic hormone (ACTH) relative to β-actin in the pituitary were determined using the Western blot method and results are expressed as mean% of control values ± SEM. *p < 0.05; **p < 0.001 compared with the control.

We examined the alterations in transcript and protein levels of POMC and ACTH in the hypothalamus and pituitary at different stages of EAE (Figure 2A). The level of *Pomc* mRNA in the hypothalamus decreased significantly at Eo when compared with naive animals (Figure 2A). In contrast, a strong induction of *Pomc* mRNA level in the anterior pituitary was demonstrated at the Eo and Ep when compared with naive animals. Next, we evaluated the protein expression pattern of POMC and ACTH in the pituitary during EAE. Densitometric analysis of Western blot revealed a significant increase in POMC and ACTH protein expression at the peak of EAE (Figure 2B).

### Histological and Stereological Parameters of Corticotrophs During EAE

**FIGURE 3:**
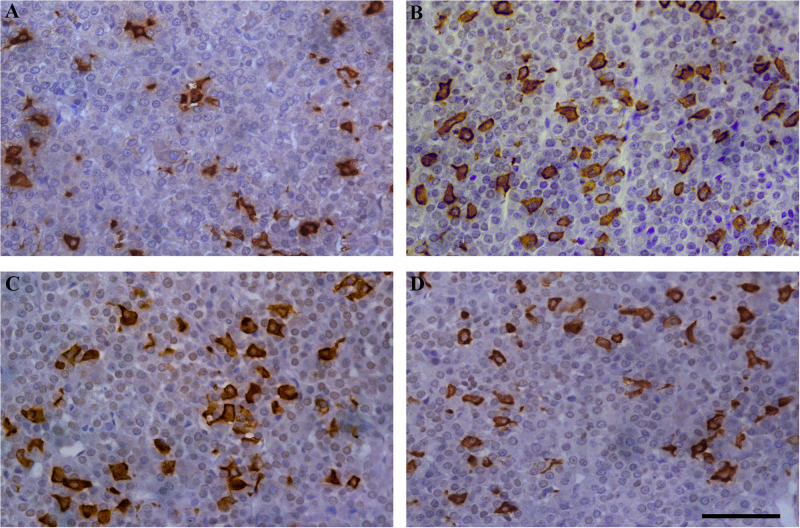
Temporal changes in the immunoreactivity of ACTH cells during EAE. Pituitary glands were obtained from rats afflicted with EAE, **(A)** control rat, and EAE rats sacrificed at onset **(B)**, peak **(C)**, and end of the disease **(D)**. Scale bar 50 μm.

Our next set of experiments aimed to investigate the pattern of tissue expression of ACTH. In all experimental animals, ACTH-immunopositive cells were irregularly shaped and located individually or in groups, between the capillaries in the pituitary *pars distalis*. Most of them were stellate, polygonal, or oval, with a round nucleus. The corticotrophs during EAE outnumbered in comparison with the control group (Figure 3).

**FIGURE 4:**
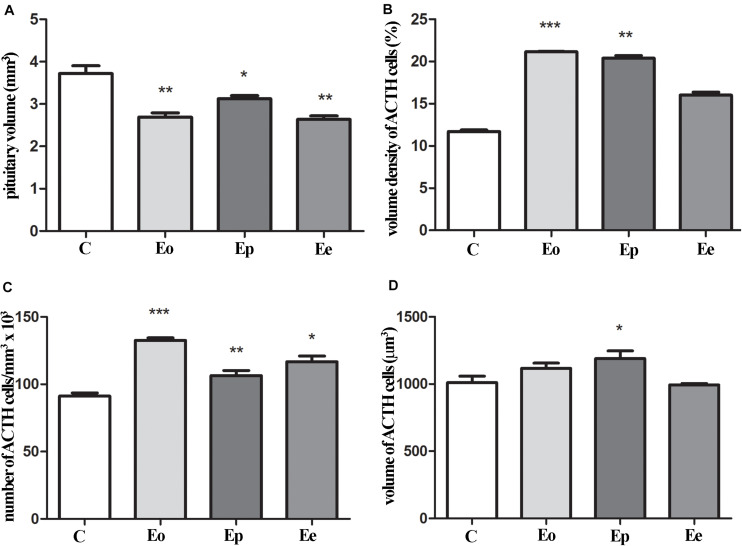
Stereological analysis of the temporal changes of ACTH cells. **(A)** Changes in the volume of the pituitary during EAE. Variation of ACTH secreting cells in the pituitary in volume density **(B)**, number **(C)**, and volume of the single cell **(D)** in the course of EAE. Pituitary from control – C and EAE rats, obtained from different time points—Eo (onset of the disease), Ep (peak of the disease), and Ee (end of the disease), and used for determination in morphometric parameters. Results are given as means ± SEM; *p < 0.05, **p < 0.01, and ***p < 0.001 on a corresponding day.

The pituitary weight and volume decreased in Eo (7.6 mg; 2.7 mm^3^) and Ep (8.8 mg; 3.1 mm^3^) in comparison with the control (9.4 mg; 3.7 mm^3^), while pituitary weight in Ee (9.2 mg; 2.6 mm^3^) (Figure 4A) tented to match the control values. The stereological analysis of the volume density of ACTH cells revealed a significant increase by 80 and 70% in Eo and Ep in comparison with the control group, respectively (Figure 4B). The number of ACTH cells increased in Eo, Ep, and Ee compared with the control values (Figure 4C). However, the volume density of a single ACTH cell shows a significant increase only in Ep when compared with the control rats (Figure 4D).

### Inflammation and Oxidative Parameters in Pituitary and Adrenal Glands During EAE

Next, we aimed at elucidating the effects of EAE in pituitary and adrenal glands of DA rats. Also, we checked the inflammation status in the serum of control and EAE animals. EAE induces a double increase in PGE2 concentration at the onset of EAE and maintains higher levels of PGE2 at Ep and Ee when compared with the control animals (Table 1). Because glucocorticoids have been closely associated with the onset and severity of EAE, we measured corticosterone serum levels as well. As expected, EAE induced a significant and almost double increase in serum corticosterone levels at the peak of EAE (983.8 ± 101.4 ng/ml) in comparison with the control animals (541 ± 94.5 ng/ml) (Table 1).

**TABLE 1:** Inflammatory and oxidative parameters during EAE.

	**Ctrl**	**Eo**	**Ep**	**Ee**
PGE2 (pg/ml)-serum	1213.2 ± 107.8	2445.0 ± 188.6**	2165.0 ± 108.7	1759.5 ± 55.7
Corticosterone(ng/ml)-serum	541 ± 94.5	595.8 ± 74.2	983.8 ± 101.4*	827.96 ± 85.8
*Tnf* (ΔCt)-pituitary	0.32 ± 0.08	0.29 ± 0.04	0.43 ± 0.03*	0.32 ± 0.05
*Il1β* (ΔCt)-pituitary	0.51 ± 0.11	0.48 ± 0.1	0.47 ± 0.06	0.26 ± 0.12
*Nos2* (ΔCt)-pituitary	0.21 ± 0.018	0.395 ± 0.06**	0.177 ± 0.09	0.159 ± 0.06
*Nfe2l2* (ΔCt)- pituitary	22.37 ± 1.92	20.3 ± 7.23	43.51 ± 5.85*	28.5 ± 6.05
*M2cr* (ΔCt)-adrenal gland	23.4 ± 2.3	30.4 ± 3.4	36.5 ± 1.9**	30.4 ± 2.7

*p < 0.05, **p < 0.01 in respect to control, Kruskal–Wallis with Dunn’s post hoc test.

**FIGURE 5:**
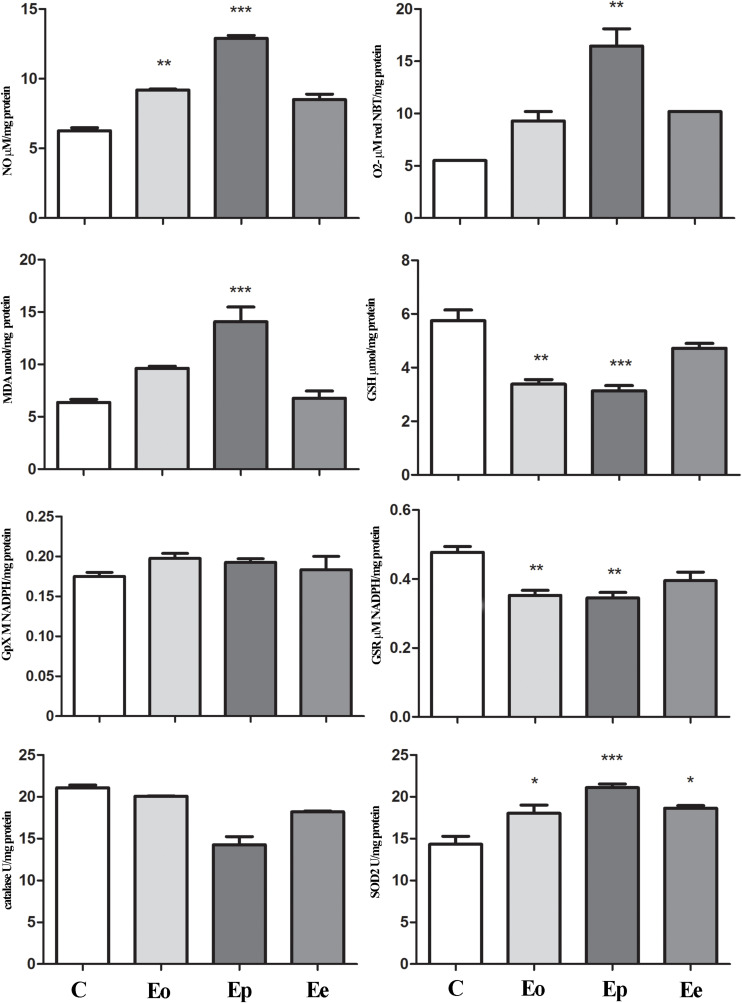
Nitrosative/oxidative status assessment in the pituitary gland during EAE. Total nitrites (NOx; μmol/mg protein) were determined to assess nitrosative status. For oxidative status assessment, O_2_^–^ (μmol/min/mg protein), GSH (μmol/mg protein), and MDA (nmol/mg protein) levels and enzymatic activities of GPx (M NADPH/mg protein), GSR (μmol NADPH/mg protein), catalase (U/mg protein), and SOD2 (U/mg protein) were determined in the pituitary gland at the onset (Eo), peak (Ep), and end (Ee) of EAE rats. Values are presented as means ± SEM. *p < 0.05, **p < 0.01, ***p < 0.001 statistically significant difference compared with the control (C) group.

*Tnf* expression was significantly upregulated at the peak of EAE in the pituitary when compared with the control animals, while *Il1*β remains unaltered throughout EAE. *Nfe2l2* expression was upregulated (twofold increase) at the peak of EAE. The increased expression of *Nos2* (almost two times in comparison with the control rats) was recorded at the onset of EAE. Afterward, the *Nos2*expression levels return to control levels (Table 1). This fact correlated with a higher NO, O_2_^–^, and lipid peroxidation rate at the onset and peak of the EAE in the pituitary (Figure 5).

**FIGURE 6:**
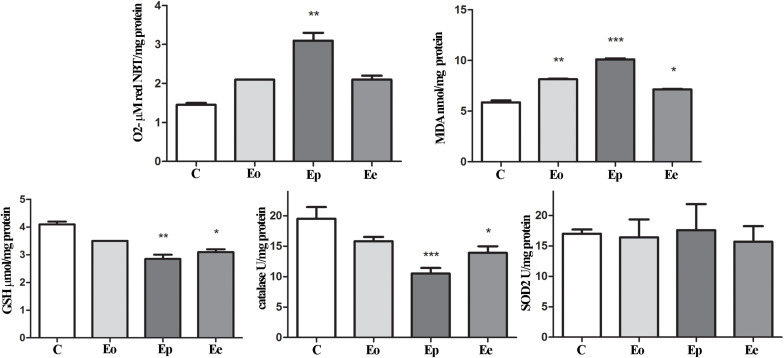
Oxidative and lipid peroxidation status assessment in the adrenal glands of rats during EAE. To assess the oxidative status, O_2_^–^ (μmol/min/mg protein), GSH (μmol/mg protein), and MDA (nmol/mg protein) levels were determined and catalase (U/mg protein) and SOD2 (U/mg protein) activities were assayed in the adrenal gland at the onset (Eo), peak (Ep), and end (Ee) of EAE. Values are presented as means ± SEM. *p < 0.05, **p < 0.01, and ***p < 0.001 statistically significant difference compared with the control (C) group.

In the adrenal glands, an upregulation of *Mc2r* expression was detected at the peak of EAE. Furthermore, a significant increase in superoxide anion and MDA that occurred during EAE was observed (Figure 6). Assessment of glutathione metabolism parameters revealed reduced levels of GSH and GSR in the pituitary at the onset and peak of EAE, while GPx levels remain unaltered during EAE (Figure 5). The GSH levels decreased in the adrenal glands during EAE (Figure 6). Catalase levels decreased at the peak of EAE in both the pituitary and adrenal glands (Figures 5, 6). Despite increased SOD2 activities in the pituitary (Figure 5), the same enzymatic activity was unaffected in the adrenal glands during EAE (Figure 6).

## Discussion

This study outlines the findings related to the activity of the HPA axis during EAE in female DA rat strain. It points and connects alterations in the pituitary and adrenal glands concerning inflammation and oxidative stress. It was previously shown that DA rats manifest a blunted HPA response, thus are prone to EAE induction (Stefferl et al., 1999). In our study, EAE developed a predictable acute monophasic course of the illness (Bjelobaba et al., 2018), accompanied by well-defined neurological impairment attributable to inflammation and demyelination (Lavrnja et al., 2008; Jakovljevic et al., 2017). Previous observations made in a mouse model of EAE revealed that PGE2 acts as a mediator of acute inflammation during the pathogenesis of EAE (Esaki et al., 2010). It was also shown that PGE2 in cooperation with cytokines may amplify their actions by making a positive feedback loop and facilitate acquired immunity-induced long-lasting inflammation (Kihara et al., 2009; Yao et al., 2009). Indeed, we here observe a strong, significant induction of PGE2 in serum at the onset of EAE, which remains high throughout the disease. The increased PGE2 levels may control the overactivated HPA axis, mainly through high corticosterone levels that act through a negative feedback loop and prevent overproduction of inflammatory molecules (Rivest, 2010).

HPA hormones have a role in the maintenance of body homeostasis, regulation of the immune system, and neuroprotection, which emphasize the importance of adequate HPA response during EAE in order to help the system to overcome disease. Previously, we have shown that EAE induces inflammation in the hypothalamus (Milosevic et al., 2020), which can activate the HPA axis. This, however, does not seem to affect gene expression in the hypothalamus. A slight decrease was observed in *Pomc*expression during EAE, as was recently shown (Tanaka et al., 2020), suggesting that the observed weight loss in EAE may not be mediated by this anorectic peptide. Feeding behavior may thus rather be inhibited by proinflammatory cytokines, like IL-1β, and TNF-α (Sarraf et al., 1997; Milosevic et al., 2020). The status of the hypothalamus also implies that the observed increase in the pituitary *Pomc* expression, as well as the protein content of POMC and ACTH, may be associated to the high levels of proinflammatory cytokines, as previously suggested (Harbuz et al., 1992). We have here shown that EAE induces the upregulation of proinflammatory cytokine *Tnf* that can further activate oxidative pathways and induce the expression of *Nos2*. Previously, we have reported increased proinflammatory cytokines and iNOS at the periphery and in the CNS tissue during EAE, all leading to massive inflammation and demyelination (Lavrnja et al., 2008; Jakovljevic et al., 2019). In our experimental settings, iNOS was upregulated in the pituitary at the onset of EAE. This may result in the observed accumulation of ROS/RNS, such as superoxide anion and nitric oxide at the symptomatic stages of EAE in the pituitary. These ROS/RNS exhibit increased levels at the onset and peak of EAE in the pituitary, returning to control levels at the end of EAE. Together, these highly reactive compounds can attack phospholipids from the membrane and cause cell damage by lipid peroxidation. Indeed, MDA levels significantly increased at the peak of EAE in the pituitary gland, pointing to amplified changes in lipoperoxidation that reflect an alteration in both structural and functional status of this organ. Lipid peroxidation involves the formation and propagation of lipid radicals, the uptake of oxygen, and the rearrangement of the double and unsaturated lipids, resulting in a variety of destroyed products that trigger devastation of membrane lipids (Ayala et al., 2014). The described results imply that pituitary corticotrophs are activated, which is confirmed by the observed increase in volume density and number of ACTH cells. The possible changes in plasma membrane integrity might take place precisely in these cells (corticotrophs). Furthermore, during EAE, the pituitary is evidently under low antioxidant status, judged by the measurements of GSH content, and GSR and CAT activity in the EAE groups is an indication of oxidative stress. Besides its other role, NADPH provides the reducing equivalents for biosynthetic reactions and the oxidation–reduction involved in the protection against ROS toxicity, by upgrading the regeneration of the main antioxidant—glutathione (Espinosa-Diez et al., 2015). A decrease of NADPH in the pituitary indicates a weakened pool of reduced equivalents necessary to provide the redox homeostasis in EAE. Interestingly, although majority of the analyzed parameters clearly indicate oxidative stress induction in the pituitary during EAE, SOD2 as a central enzyme of antioxidant defense system (Birben et al., 2012) showed an increase in activity at the peak of EAE. Literature data have shown that increased SOD2 levels might be mediated by the nuclear factor (erythroid-derived-2)-like 2 factor (Nrf2) pathway (Miljković and Spasojević, 2013). The Nrf2 pathway is a critical mechanism that upregulates antioxidative enzymes and proteins directed toward redox restitution (Kobayashi et al., 2004). It was shown that the absence of Nrf2 aggravates EAE (Johnson et al., 2010), inducing oxidative stress development. In line with that view, Nrf2 upregulation is considered to be responsible for restored oxidative balance in the pituitary gland after EAE induction (Spiers et al., 2015). We have shown its upregulation at the mRNA level in the pituitary at the peak of EAE. Literature data have shown that mitochondrial ROS may activate Nrf2, usually through protein kinases, inducing the expression of antioxidant genes, but also genes controlling mitochondrial quality/quantity (Kasai et al., 2020).

Previous studies showed that EAE increased ACTH levels which activates the Mc2r in the zona fasciculata of the adrenal cortex (Smith and Vale, 2006) and subsequently promotes *de novo* synthesis and release of corticosterone (Harbuz et al., 1993; Spiga et al., 2011). In this study, we also demonstrate the increased *Mc2r* expression in the adrenal glands at the peak of EAE accompanied by increased serum levels of corticosterone at the peak of EAE, as was previously shown in other strains of animals (Stefferl et al., 1999; Esquifino et al., 2004). Endogenous corticosterone inhibits innate and adaptive responses, mainly through reducing lymphocyte proliferation and inhibiting cytokine and antibody production (Webster Marketon and Glaser, 2008), thus ameliorating the clinical score of EAE (Sorrells and Sapolsky, 2007). Indeed, adrenalectomy in EAE induced massive peripheral inflammation, underpinning the importance of circulating glucocorticoids in preventing inflammation (Mason et al., 1990; Smith et al., 1996). High plasma cortisol levels and elevated HPA activation were also recorded in MS patients (Melief et al., 2013). Admittedly, it was proposed that in MS, the sensitivity of immune cells to glucocorticoids is impaired, most prominently in patients with the relapsing-remitting disease (Gold et al., 2012).

While many studies were centered on the role of glucocorticoids in MS/EAE, the knowledge on the status of adrenal gland tissue is still quite limited. The adrenal cortex is endowed with high levels of enzymatic and non-enzymatic antioxidants, which are able to handle the enhanced risk factors for oxidative stress. During EAE, we have shown increased O_2_^–^ production and MDA concentration at the peak of EAE, implying changes at the membrane of cells in the adrenal gland. Also, GSH content and catalase activity decreased at the peak and end of the EAE compared with control values, while no significant change between the groups was observed considering SOD2 activity. The glutathione redox cycle merges GSH and GSH-associated enzymes and has a crucial antioxidative defense role in neuronal tissue (Forman et al., 2009). EAE induction significantly reduced GSH content at the peak and end of EAE compared with controls. Glutathione scavenges free radicals through non-enzymatic and/or enzymatically catalyzed reactions when it becomes converted immediately into its disulfide form, GSSG, which becomes efficiently reduced back to GSH (Forman et al., 2009).

## Conclusion

This study revealed a strong imbalance in the redox system in pituitary and adrenal glands, which may be involved in the observed activation of the HPA axis obtained in our experiments. The HPA activation was followed by an increased number and volume of corticotrophs in the pituitary at the peak of EAE. The increased gene and protein levels of POMC and ACTH in the pituitary lead to increased corticosterone levels. We may conclude that HPA activation during EAE is associated with the increase of ROS at the pituitary and adrenal levels.

## Data Availability Statement

The raw data supporting the conclusions of this article will be made available by the authors, without undue reservation.

## Ethics Statement

The animal study was reviewed and approved by the Ministry of Agriculture, Forestry and Water Economy of the Republic of Serbia (permit no. 323-07-05970/2020-05).

## Author Contributions

ST, NR, DL, and IL conceived and designed the experiments. IS, AM, MJak, IBo, IBj, DS, and KT performed the experiments. ST, IS, MJan, and IL analyzed the data. MJan, IBj, and IL contributed to the writing of the manuscript. All authors contributed to different aspects of this work, such as the experimental design and the acquisition, analysis, and interpretation of data; finally approved the submitted version of the manuscript; and agreed to be amenable to all aspects of the work.

## Conflict of Interest

The authors declare that the research was conducted in the absence of any commercial or financial relationships that could be construed as a potential conflict of interest.
